# Efficacy of Telepsychiatry in Refugee Populations: A Systematic Review of the Evidence

**DOI:** 10.7759/cureus.3984

**Published:** 2019-01-30

**Authors:** Ahmad Hassan, Kareem Sharif

**Affiliations:** 1 Psychiatry, Yale University School of Medicine, New Haven, USA; 2 Psychiatry, University of Pennsylvania School of Medicine, Philadelphia, USA

**Keywords:** syrian refugees, telemedicine, telepsychiatry, trauma, post-traumatic stress disorder (ptsd)

## Abstract

Introduction

Telepsychiatry is becoming an increasingly appealing option for mental health treatment due to its ability to overcome barriers which prevent certain demographics from having access to mental health services. There is a surprising lack of research being done on this promising mode of health care delivery. The aim of this study is to evaluate the existing literature in order to determine the clinical effectiveness and cost-effectiveness of telepsychiatry in resource-constrained environments.

Methods

Literature searches were performed in PsychINFO, PubMed, Medline, EMBASE, Centre for Reviews and Dissemination, and the Cochrane Library Controlled Trial Registry databases (2000 - May 2017). A search of the following terms was used: telemedicine; telemedical; telepsychiatry; telepsychiatric; teleconsultation; e-health; video conference; and telecare. Type of mental disorder and intervention, along with the clinical outcome or patient satisfaction, were all identified. Exclusion criteria included studies with a sample size of fewer than 10 cases, as well as studies which failed to analyze intervention outcomes.

Results

Of the 1,477 identified articles, 14 randomized controlled trials were included for review. Despite the methodological limitations and the small number of existing studies, there appears to be limited evidence pointing towards the efficacy of telepsychiatry in resource-constrained environments, although patients and providers tend to prefer face-to-face treatment over video conferencing. Two of the studies included in this paper found video conferencing to be more effective than face-to-face treatment, while none reported the opposite. At the very least, we hypothesize that psychotherapeutic treatment delivered via video conferencing is just as effective as a traditional treatment, albeit less desirable.

Conclusion

More research is required in order to further evaluate the efficacy of telepsychiatry in the management of mental illness, as there is a current lack of scientific evidence to draw any conclusions. However, there exists a strong hypothesis that telepsychiatric treatment yields the same results as the traditional, in-person therapy and that telepsychiatry is a useful alternative when traditional therapy is not possible. Countries with substantial numbers of refugees living in resource-constrained areas, such as camps, should be encouraged to develop telepsychiatry programs.

## Introduction

It has currently been more than seven years since the advent of the Syrian conflict, and the number of Syrian citizens displaced internally, along with refugees who are now located in neighboring countries, has reached staggering numbers. The United Nations High Commissioner for Refugees (UNHCR) has identified approximately 13.5 million Syrians in need of humanitarian assistance, of which 5 million are refugees outside of the country and 6 million are internally displaced [1]. It is estimated by the Turkish government authorities that more than half of the Syrian refugees are in dire need of mental health treatment and psychiatric services; yet, they remain untreated [2]. Merely 5% of the necessary psychiatric services are provided for refugees in Syria, Lebanon, and Turkey [3]. One of the biggest obstacles preventing Syrian refugees from receiving psychiatric treatment is a language barrier, as the majority of Syrian refugees only speak Arabic. There is a severe shortage of Arabic-speaking mental health professionals in the region due to the lack of educational programs offering certification. Additional obstacles include access to refugees and the high costs of hiring mental health professionals to stay in the area and follow-up with patients [4]. The sheer amount of Syrian refugees entering neighboring countries puts pressure on the healthcare system and economy of countries already suffering from weak services and infrastructure [5].

In March of 2016, Amnesty International issued a report regarding the dire situation of Syrian refugees residing in Jordan and other neighboring countries. They stated that of the several million Syrian refugees residing in neighboring countries, only a mere 117,000 of them live in camps where health care, food, water, and education are readily accessible [1]. Meanwhile, the vast majority of refugees currently residing in neighboring countries live outside of these refugee camps and need government documents in order to access public services. Refugees who have left a refugee camp unofficially (oftentimes due to the horrid environment) are ineligible to receive these government documents, leaving them without access to public services and completely reliant on humanitarian aid. In November of 2014, the Jordanian government implemented fees for refugees in order for them to access subsidized medicines, which pose a heavy burden as over 90% of Syrian refugees are already living below the poverty line [3].

It is becoming more evident that telepsychiatry is one of the only treatment options which can be installed in the near future that manages to overcome all of these barriers, while still providing adequate treatment to those suffering from psychopathologies. A recent study found that telepsychiatry is equally as effective as regular treatment in the case of treating patients with post-traumatic stress disorder (PTSD). The study involved veterans who were deployed in Iraq and Afghanistan and suffered from PTSD and analyzed the effectiveness of cognitive processing therapy received through video calling [6]. Given the apparent effectiveness of telepsychiatry, along with the lack of adequate mental health treatment options available to Syrian refugees, it is necessary to determine whether those in resource-constrained environments would benefit from telepsychiatry. The objective of this review is to evaluate the feasibility and efficacy of telepsychiatry in populations living in resource-constrained environments, so that it may be determined whether a large enough evidence base supports the efficacy telepsychiatry among refugee populations.

## Materials and methods

Since as early as 1957, experts have been researching and producing publications on telepsychiatry. However, the literature of telepsychiatry is generally characterized by poor descriptive evaluation studies and very few quality evidence studies analyzing the efficacy of telepsychiatry. Additionally, only a few papers analyzing the efficacy of telepsychiatry in the developing world have been published. It is, therefore, necessary to evaluate the existing literature on telepsychiatry implementation before considering the implementation of telepsychiatry in Syrian refugee camps. Similar studies have been conducted previously but are either outdated or do not filter for specifically high-quality randomized controlled trial (RCTs). 

The first step was to identify whether there were any existing RCTs detailing the efficacy of telepsychiatry published in the literature. The systematic search that was undertaken accessed published articles from 2000 onwards. Before the systematic search was conducted, an initial, limited search of PubMed and Medline was undertaken to determine relevant keywords contained in the title, abstract, and subject descriptors. This allowed the reviewers to determine MeSH terms and synonyms used by respective databases which were then utilized in an extensive search of the literature. Computerized literature searches were performed in PsycINFO, Medline, PubMed, EMBASE, Centre for Reviews and Dissemination, and The Cochrane Library Controlled Trial Registry databases (2000 – May 2017), in addition to a manual search of systematic reviews and meta-analyses. The search terms were: telemedicine; telemedical; telepsychiatry; telepsychiatric; teleconsultation; e-health; video conference; and telecare. In PubMed, the search terms used were: telemedicine (MeSH term) AND psychiatry (MeSH term) AND review.

**Figure 1:**
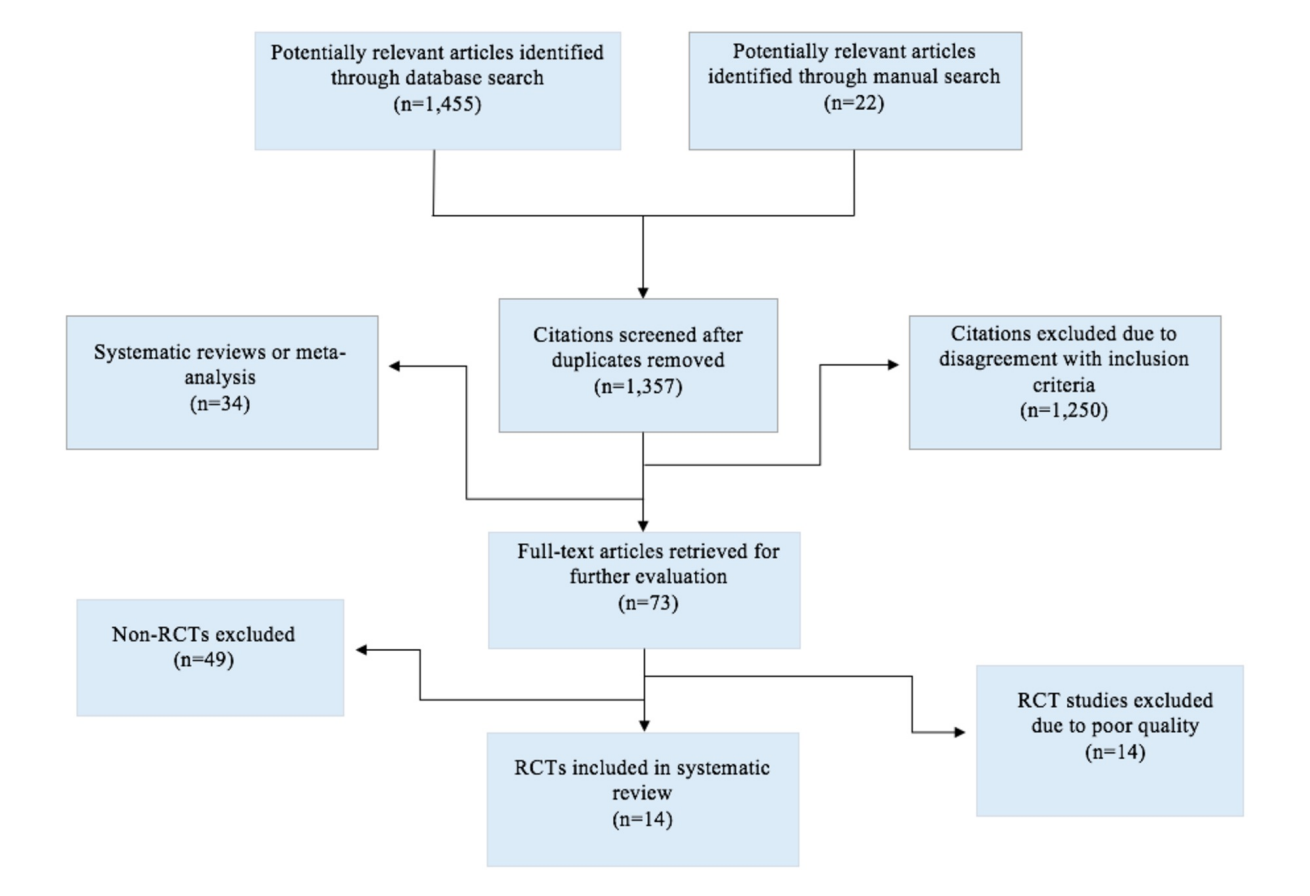
Systematic Review Retrieval Process RCT = randomized controlled trial

The articles considered for inclusion in this review included articles where: (1) the study design was an RCT; (2) participants consisted of patients with a diagnosable mental disorder (according to the Diagnostic and Statistical Manual of Mental Disorders (DSM-IV) definitions); (3) the study included information on patient satisfaction or clinical outcomes; and (4) the use of video conferencing or televideo technology was used. Exclusions included studies published before the year 2000, studies that utilized the internet and phone-based telepsychiatry, and studies that did not evaluate intervention outcomes. Articles were initially screened based on their abstracts, and studies without an abstract were excluded. Two reviewers then thoroughly and independently examined the studies which satisfied the inclusion criteria in order to assess the study quality and avoid redundancies. The retrieval process is summarized in Figure 1.

## Results

One thousand four hundred and seventy-seven (1,477) article abstracts on general telepsychiatry were retrieved from the year 2000 onwards (Figure 1). One hundred and twenty articles were duplicates (120), 34 were excluded due to being systematic reviews or meta-analyses, and 1,250 were excluded due to disagreement with inclusion criteria. Seventy-three full-text articles were suitable for full paper retrieval based on review methodology. Forty-nine (49) of these papers were removed due to being non-RCTs, and 14 RCTs were excluded due to poor quality according to the Cochrane recommendations. Of these, 14 RCTs that met the study quality and inclusion criteria were selected for detailed extraction of findings and evidence-based recommendations (Figure 1). The characteristics of the 14 RCTs are described in Table 1.

**Table 1:** Randomized Controlled Trials Included in Systematic Review The intervention group received treatment through video conferencing. The control group received traditional face-to-face (FTF) treatment. Abbreviations: CBT = cognitive behavioral therapy; N/A = not available; NSp = not specified,

Study	Country	Type of Disorder	Sample Size, (intervention/control)	Age, y (follow-up)	Intervention	Results
Elford et al., 2000 [7]	Canada	Multiple	23 (12/13)	< 18 (3 mo.)	Objectives: Education and treatment; Program: NSp; Duration: NSp	Patient satisfaction: Most were satisfied, the majority preferred FTF; Parent satisfaction: Most were satisfied; Therapist satisfaction: Satisfied but preferred FTF
Nelson et al., 2003 [8]	United States	Depression	28 (14/14)	8-14 (8 wk.)	Objectives: Diagnosis and treatment; Program: CBT; Duration: 8 wk.	Patient satisfaction: High; Symptoms: Better in intervention group (P < .05)
Cuevas et al., 2006 [9]	Spain	Multiple	140 (70/70)	> 18 (NSp)	Objectives: Treatment; Program: CBT and prescription; Duration: 24 wk.	Satisfaction: NSp; Efficacy: Just as effective as FTF treatment (p < .001).
Mitchell et al., 2008 [10]	United States	Bulimia Nervosa	128 (62/66)	> 18 (1 yr.)	Objectives: Treatment and psychoeducation; Program: CBT; Duration: 16 wk.	Patient satisfaction: Generally satisfied; Efficacy: Roughly equivalent in outcome to FTF treatment.
Hilty et al., 2007 [11]	United States	Depression	94 (47/47)	> 18 (1 yr.)	Objectives: Diagnosis and treatment; Program: CBT; Duration: 18 wk.	Symptoms: Significant improvement; Patient satisfaction: High
Chong et al., 2012 [12]	United States	Depression	167 (80/87)	> 18 (NSp)	Objectives: Treatment; Program: Psychoeducation; Duration: 6 mo.	Efficacy: No differences were found in overall depression score between intervention and control; Patient satisfaction: NSp
Spaniel et al., 2015 [13]	United States	Schizophrenia	146 (74/72)	> 18 (NSp)	Objectives: Treatment; Program: Relapse prevention program; Duration: 18 mo.	Patient satisfaction: NSp; Therapist satisfaction: NSp
Frueh et al., 2007 [14]	Canada	PTSD	38 (17/21)	> 18, Mean: 56 (3 mo.)	Objectives: Treatment; Program: CBT; Duration: 14 wk.	Patient satisfaction: NSp; Therapist satisfaction: NSp; Adherence to treatment: Better in control group (P = .04)
Ruskin et al., 2004 [15]	United States	Depression	119 (59/60)	> 18, Mean: 49.7 (6 mo.)	Objectives: Treatment; Program: Psychoeducation; Duration: 26 wk.	Patient satisfaction: NSp; Therapist satisfaction: Higher in control group; Cost: Lower in control group (P < .001)
O’Reilly et al., 2007 [16]	Canada	Multiple	495 (241/254)	> 18 (12 mo.)	Objectives: Diagnosis and follow-up; Program: Treatment management; Duration: 4 mo.	Patient satisfaction: No difference between intervention and control; Therapist satisfaction: No difference between intervention and control; Cost: Lower in intervention group
Bishop et al., 2002 [17]	Canada	Multiple	24 (11/10)	> 18 (4 mo.)	Objectives: Diagnosis and follow-up; Program: NSp; Duration: 4 mo.	Patient satisfaction: No difference between intervention and control; Therapist satisfaction: No difference between intervention and control
Bouchard et al., 2004 [18]	Canada	Panic disorder	21 (11/10)	> 18 (6 mo.)	Objectives: Treatment; Program: CBT, psychoeducation; Duration: 12 wk.	Patient satisfaction: NSp; Therapist satisfaction: NSp; Efficacy: More improvement was seen in intervention group (P < .05)
Manguno-Mire et al., 2007 [19]	United States	Mental Incompetency	21 (N/A)	> 18 (NSp)	Objectives: Evaluation of mental competency to determine fitness to stand trial	Patient satisfaction: Patients did not express a preference for one over the other; Therapist satisfaction: Greater satisfaction was reported for FTF interviews; Efficacy: High levels of agreement between telemedicine and live interviews
Fortney et al., 2013 [20]	United States	Depression	395 (177/218)	> 18, Mean: 59 (Measured at 6-month and 12-month checkpoints)	Objectives: Treatment; Program: Treatment and psychoeducation; Duration: 1 yr.	Efficacy: intervention group was more likely to be adherent at both 6 and 12 months; also reported larger gains in mental health status

Eight trials were conducted in the United States [8, 10-13, 15, 19-20], five were conducted in Canada [7, 14, 16-18], and one was conducted in Spain [9]. The RCTs included in this study encompassed a wide range of mental illnesses, including depression, panic disorder, PTSD, schizophrenia, and bulimia nervosa, all of which are disorders commonly seen in Syrian refugee patients. In total, 1,714 patients were included in all of the RCTs selected for this study. Generally, each RCT consisted of one intervention group receiving treatment through video conferencing and one control group receiving traditional face-to-face (FTF) therapy. Several studies used cognitive-behavioral therapy [8-11, 14, 18], while the majority did not specify the exact type of psychotherapy used. It is worth noting that one study did not involve psychotherapy, and instead studied the efficacy of video conferencing in evaluating whether a convicted criminal was fit to stand trial [19].

Only five studies [8-10, 12, 18] evaluated and compared the symptoms of patients in the intervention and control groups. Of these, only Nelson et al. [8] and Bouchard et al. [18] found a greater improvement of symptoms among patients in the intervention group. In addition, Cuveas et al. [9], Mitchell et al. [10], and Chong et al. [12] found no difference in symptoms among the intervention and control groups. None of the studies reported a greater improvement in symptoms among patients in the control group compared to the intervention group.

Seven studies [7-8, 10-11, 16-17, 19] directly evaluated the satisfaction of patients with video conferencing and traditional FTF treatments. Elford et al. [7], Nelson et al. [8], and Hilty et al. [11] found that patients reported high levels of satisfaction with video conferencing, although Elford et al. [7] found that the majority of the patients still preferred FTF treatment. Mitchell et al. [10] reported lower levels of patient satisfaction with video conferencing. O’Reilly et al. [16], Bishop et al. [17], and Manguno-Mire et al. [19] reported no difference in the patient level of satisfaction between intervention and control groups.

Five studies [7, 15-17, 19] evaluated the level of satisfaction of therapists administering treatment. Elford et al. [7] found that although therapists were generally satisfied with video conferencing, they preferred FTF treatment. O’Reilly et al. [16] and Bishop et al. [17] found no difference between therapists’ level satisfaction between video conferencing and traditional FTF treatment. Ruskin et al. [15] and Manguno-Mire et al. [19] found that therapists reported higher levels of satisfaction in delivering FTF treatment as opposed to video conferencing. 

## Discussion

None of the RCTs included in this study were conducted in developing countries or in resource-constrained environments, which calls into question the degree to which the evidence-based recommendations generated by these studies apply to resource-constrained environments, such as Syrian refugee camps. To address potential biases in the application of these recommendations, it is critical that only evidence of the highest quality is applied to other settings. Until RCTs evaluating the efficacy of telepsychiatry are conducted in developing countries, evidence must be drawn from the available literature and applied to other settings very carefully.

The results of this systematic review show that there is limited evidence supporting the efficacy of telepsychiatry programs, but in agreement with several authors cited in this study, we believe all data pointing towards telepsychiatry as being feasible and effective. Considering that two of the studies found video conferencing to be more effective than FTF treatment, while none reported the opposite, it can be reasonably concluded that psychotherapeutic treatment delivered via video conferencing is just as effective as traditional treatment. Although the majority of studies showed that patients and therapists generally were more satisfied and preferred FTF treatment, Syrian refugees living in camps are in dire need of any form of treatment at all, and video conferencing seems to be an adequate mode of delivery considering its apparent effectiveness.

It is hypothesized that telepsychiatry is equally as effective as traditional FTF treatment in the majority of situations. The reason for its effectiveness is because this method of treatment overcomes several major obstacles, notably those of cost, provider access to patients, and social stigma surrounding mental health intervention. One study, in particular, supporting the hypothesis that telepsychiatry is equally as effective as traditional therapy was conducted by Maieritsch et al. [6]. This study was performed with Iraq and Afghanistan veterans suffering from PTSD and found evidence to support that telepsychiatry is equally as effective as traditional treatment. The study primarily studied the efficacy of cognitive processing therapy conducted via video conferencing, and this method of treatment was found to be equally as effective as a standard treatment delivered in person [6]. 

As for the cost-effectiveness of telepsychiatry, it is hypothesized that video conferencing reduces the cost of delivering psychotherapeutic treatment only in resource-constrained environments. The majority of existing literature points to telepsychiatry having lower direct and indirect costs than traditional FTF treatment. Although telepsychiatry has greater up-front costs associated with setting up the appropriate equipment for delivery, it is believed that eventually, as the number of consultations increase, telepsychiatry becomes more cost-effective than FTF therapy [21]. While the number of consultations at which telepsychiatry becomes more cost-effective than FTF therapy differs based on the context of each study, it is worth noting that in rural areas, only a few consultations are needed before the cost-effectiveness of telepsychiatry is seen. Two studies have seen telepsychiatry to be more expensive than traditional treatment, but this may be due to small sample size or to not following up with enough consultations [22-23]. 

There are currently very little studies evaluating the feasibility and cost of telepsychiatry. Although one of the RCTs included in this study reported lower costs in the control group [15], other existing studies have reported the opposite. We hypothesize that telepsychiatry is a cost-effective method of treatment only in resource-constrained environments. More RCTs evaluating the efficacy and feasibility of telepsychiatry in resource-constrained environments and otherwise are needed to comprehensively and accurately evaluate the effectiveness of telepsychiatry programs implemented in Syrian refugee camps. These studies should measure patient and professional satisfaction, as well as standard patient outcomes, along with the quality of care, accessibility to treatment, and cost. 

## Conclusions

Although there is currently insufficient evidence to reasonably determine the effectiveness of telepsychiatry, countries that contain a high number of resource-constrained settings, such as Syrian refugee camps (including Lebanon, Jordan, and Turkey), are encouraged to develop telepsychiatry programs and appropriate evaluation strategies, in addition to supporting research to increase the amount of existing literature and evidence regarding the effectiveness of telepsychiatry. We strongly believe that telepsychiatric treatment is equally as effective as FTF treatment in the majority of contexts and is particularly useful in environments where FTF therapy is not an option. The implementation of telepsychiatry into Syrian refugee camps will almost certainly improve the quality and amount of mental health treatment provided to refugees. Large-scale implementation of video conferencing technology will not only ease the economic burden on neighboring countries in the long-term but will provide millions of refugees with access to adequate mental health treatment who otherwise would not receive it. One of the major obstacles preventing refugees from receiving treatment is the lack of Arabic-speaking mental health professionals in the region due to a small number of programs producing certified specialists, in addition to the lack of incentives provided to specialists who choose to work in refugee camps. Implementing video conferencing technology would certainly provide refugees with access to Arabic-speaking mental health professionals, and while the full potential of telepsychiatry would take several years after implementation to be realized, there are many potential benefits which can be realized in the short-term. 
